# Knockdown of KLF5 ameliorates renal fibrosis in MRL/lpr mice via inhibition of MX1 transcription

**DOI:** 10.1002/iid3.937

**Published:** 2023-07-27

**Authors:** Shanshan Tao, Xiao Tan, Wen Chai, Xiaojie Peng, Weimin Zheng, Rui Fu, Meihui Deng

**Affiliations:** ^1^ Department of Nephrology, Jiangxi Provincial Children's Hospital The Affiliated Children's Hospital of Nanchang University Nanchang Jiangxi China; ^2^ Department of Hematology, Jiangxi Provincial Children's Hospital The Affiliated Children's Hospital of Nanchang University Nanchang Jiangxi China; ^3^ Department of Neurology, Jiangxi Provincial People's Hospital The First Affiliated Hospital of Nanchang Medical College Nanchang Jiangxi China

**Keywords:** KLF5, lupus nephritis, MRL/lpr mouse, myxovirus resistance 1, renal fibrosis

## Abstract

**Objective:**

This study aims to elucidate the role of Kruppel‐like factor (KLF5) and myxovirus resistance 1 (MX1) in the progression of renal fibrosis in lupus nephritis (LN).

**Methods:**

First, the expression of KLF5 and MX1 was assessed in the peripheral blood of LN patients and healthy participants. Next, the pathological changes in renal tissues were evaluated and compared in BALB/c and MRL/lpr mice, by detecting the expression of fibrosis marker proteins (transforming growth factor‐β [TGF‐β] and CTGF) and α‐SMA, the content of urine protein, and the levels of serum creatinine, blood urea nitrogen, and serum double‐stranded DNA antibody. In TGF‐β1‐induced HK‐2 cells, the messenger RNA levels of KLF5 and MX1 were tested by qRT‐PCR, and the protein expression of α‐SMA, type I collagen (Col I), fibronectin (FN), and matrix metalloproteinase 9 (MMP9) was measured by western blot analysis. Moreover, the relationship between KLF5 and MX1 was predicted and verified.

**Results:**

In renal tissues of MRL/lpr mice and the peripheral blood of LN patients, KLF5 and MX1 were highly expressed. Pearson analysis revealed that KLF5 was positively correlated with MX1. Furthermore, KLF5 bound to MX1 promoter and promoted its transcription level. MRL/lpr mice showed substantial renal injury, accompanied by increased expression of α‐SMA, TGF‐β, CTGF, Col I, FN, and MMP9. Injection of sh‐KLF5 or sh‐MX1 alone in MRL/lpr mice reduced renal fibrosis in LN, while simultaneous injection of sh‐KLF5 and ad‐MX1 exacerbated renal injury and fibrosis. Furthermore, we obtained the same results in TGF‐β1‐induced HK‐2 cells.

**Conclusion:**

Knockdown of KLF5 alleviated renal fibrosis in LN through repressing the transcription of MX1.

## Graphical abstract

1. MX1 was highly expressed in LN mice; 2. Downregulation of MX1 relieved renal fibrosis in LN mice; 3. The expression of KLF5 was increased in LN mice; 4. KLF5 positively regulated MX1 transcription; 5. Knockdown of KLF5 mitigated renal fibrosis in LN by inhibiting MX1 transcription.

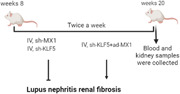

## 1 INTRODUCTION

Systemic lupus erythematosus (SLE) is an autoimmune connective‐tissue disorder, which often lead to complication in organs and tissues, most frequently in the kidney.^1^, ^2^ The central pathogenic mechanisms of SLE are the generation of pathogenic autoantibodies and type Ⅰ interferon signaling.^3^ Lupus nephritis (LN) is one of the most serious manifestations of SLE,^4^ which occurs in approximately 50% of patients with SLE.^5^ The onset and development of LN is the result of intricate interactions between immune response control and pathological process involving renal resident cells, which affects a wide spectrum of kidney structure.^6^ Disappointedly, many LN patients with the application of anti‐inflammatory and immunosuppressive therapies still developed chronic or end‐stage renal diseases.^7^ Hence, a deeper understanding of the molecular mechanisms in LN is required for the development of new treatment options for LN patients.

Myxovirus resistance 1 (MX1) is an interferon‐induced gene that encodes a GTPase and plays an important role in the defense of mammalian cells against influenza A virus and other viruses.^8^ Previously, MX1 has been studied in autoimmune diseases. For example, MX1 level was used as a routine marker for the assessment of primary Sjögren's syndrome (pSjS) disease activity.^9^ MX1 was one of the top 10 differentially expressed genes in patients with SLE,^10^ and a former study directly pointed out higher MX1 expression level in SLE patients with LN than in those without LN.^11^ Additionally, MX1 expression was upregulated in pristine‐induced lupus mice.^12^ However, the role of MX1 in renal fibrosis in LN remains largely unknown.

Most members of the Kruppel‐like transcription factor family have been shown to modulate the physiologic process in the kidney, from maintaining glomerular filtration barrier to tubulointerstitial inflammation to progression of renal fibrosis, including KLF5.^13^ A previous study reported that KLF5 was related to the regulation of renal fibrosis, tubulointerstitial inflammation, podocyte apoptosis, and renal cell proliferation.^14^ KLF5 was found to be upregulated in renal tissues of mice with diabetic nephropathy (DN), and inhibited KLF5 could relieve podocyte injury, which may be helpful for establishing a novel DN therapy.^15^ Li ZL et al.^16^ demonstrated that KLF5 deficiency could reduce the expression of transforming growth factor‐β1 (TGF‐β1) in high‐dose MK‐8617‐treated HK‐2 cells and alleviate renal fibrosis. Current evidence suggested that KLF5 is an important regulator of renal disease, but its role in clinical nephritis has not been investigated. Furthermore, the prediction results from the JASPER database (https://jaspar.genereg.net/) in our work displayed that KLF5 can bind MX1. In addition, MRL/lpr mouse is a common used mouse model for LN research for 16‐week aged MRL/lpr mouse can develop lupus‐like syndromes similar to human SLE.^17^ Therefore, we used MRL/Lpr mice as a representative animal model for our study. Based on the above evidence, we hypothesized that KLF5 may affect renal fibrosis and injury in MRL/lpr mice by regulating MX1 transcription. Consequently, this work was designed to verify this hypothesis, in an attempt to provide a new therapeutic insight for LN.

## 2 MATERIALS AND METHODS

### 2.1 Gene Expression Omnibus (GEO) database analysis of differentially expressed genes (DEGs)

First, LN associated gene expression chips were searched in GEO database (https://www.ncbi.nlm.nih.gov/geo/) with the key word LN. Next, GSE32591 and GSE112943 chips were used for subsequent analysis. Renal tissue samples were analyzed by the limma method using SangerBox software with |logFC| > 1 and adjust *p* < .05 as the standard screening thresholds. The DEGs were displayed by a volcano plot.

### 2.2 Clinical samples

According to International Society of Nephrology/Renal Pathology Society (ISN/RPS) 2003 classification of LN,^18^ 30 patients (5 type II, 5 type III, 5 type IV, 5 type V, 5 type V–III, and 5 type V–IV) who were diagnosed as LN in Jiangxi Provincial Children's Hospital were selected as the LN group (12 men and 18 women, aged 42.1 ± 8.8 years). Patients were included if they matched the following criteria: (1) diagnosed as SLE; (2) diagnosed as LN by renal biopsy results; (3) complete clinical and pathological data. Patients were excluded if they match the following criteria: (1) patients had the symptoms of diabetes mellitus, HBV infection, hepatitis, or malignancy tumors; (2) patients received continuous renal replacement therapy; (3) women were undergoing pregnancy or breastfeeding. Meanwhile, 30 healthy participants who underwent physical examination at the same time were selected as the control group (14 men and 16 women, aged 41.6 ± 9.1 years). There was no significant difference in age, sex, and other general data between LN and control groups (*p* > .05). Fasting venous peripheral blood (5 mL) was collected from each group. The anticoagulated blood was centrifuged before the sublayer blood was collected and stored at −20°C. All participants provided written informed consent, and the study was approved by the Clinical Research Ethics Committee of Jiangxi Provincial Children's Hospital.

### 2.3 Animal

Forty female MRL/lpr mice and eight female BALB/c mice (all 7 weeks old; Nanjing Junke Bioengineering Co., Ltd.) were fed in specific pathogen free circumstances at 22°C–25°C and 55%–65% humidity for 1 week. Artificial light‐dark was 12 h:12 h with the light intensity of 200–300 lx. The air change frequency was 20 times/h and the ammonia concentration was below 14 mg/m^3^. Sterile water and 60Co irradiated pellet feed were provided by Shanghai SLAC Laboratory Animal Co., Ltd.

### 2.4 Animal grouping and treatment

The adenovirus vectors of KLF5 knockdown (sh‐KLF5), MX1 overexpression (ad‐MX1), MX1 knockdown (sh‐MX1), and their negative controls (ad‐NC, sh‐NC) were purchased from GenePharma. MRL/lpr mice were classified into five groups with eight mice in each group: MRL/lpr group, sh‐MX1 (mice were injected with sh‐MX1) group, sh‐NC (mice were injected with sh‐NC) group, sh‐KLF5 (mice were injected with sh‐KLF5) group, and sh‐KLF5 + ad‐MX1 (mice were injected with sh‐KLF5 + ad‐MX1) group. After adaptive feeding for 1 week, mice in the sh‐MX1, sh‐NC, sh‐KLF5, and sh‐KLF5 + ad‐MX1 groups were injected with adenovirus vectors (1 × 10^9^ pfu/100 μL) through their tails for 12 weeks, twice a week. When the mice were 20 weeks old, they were anesthetized by intraperitoneal injection of 1% pentobarbital sodium (80 mg/kg) and then euthanized by cervical dislocation. Blood was extracted from the right atrium with a 1.0 mL syringe, and centrifuged to separate serum for subsequent experiments. Immediately, the renal tissues of mice were dissected on the ice platform. Part of the renal tissues was used for pathological observation, and the rest were frozen at −80°C for quantitative reverse transcription‐polymerase chain reaction (qRT‐PCR) and western blot analysis. The project was approved by the Animal Ethics Committee of Jiangxi Provincial Children's Hospital, and all animal experiments were performed in accordance with the Guidelines for the Care and Use of Experimental Animals approved by the International Committee.

### 2.5 Determination of urine protein, serum creatinine, urea nitrogen, and double‐stranded DNA (dsDNA)

Urine of mice 24 h before euthanization was collected by micturition reflex method, and coomassie brilliant blue G250 was used to measure protein content in urine according to the instructions of protein quantification kit (Boster Biological Technology Co. Ltd.). The optical density value was measured with a microplate reader at a wavelength of 595 nm, and the protein concentration of the sample was calculated according to the standard curve. The serum of 20‐week‐old mice was obtained, in which the content of serum creatinine and urea nitrogen were measured by an automatic biochemical analyzer. The level of dsDNA was detected by mouse anti‐dsDNA antibody enzyme‐linked immunosorbent assay kit.

### 2.6 Hematoxylin‐eosin (H&E) staining

Renal tissue was fixed in 4% paraformaldehyde for 48 h and then cut into 4‐μm paraffin sections. Sections were dewaxed and rinsed in running water. After that, the sections were stained by hematoxylin for 3–5 min, followed by rinsing, differentiation, and treatment with ammonium hydroxide. Next, the sections were stained with eosin for 5 min after dehydration with 85% and 95% alcohol for 5 min each, after which the sections were dewaxed, dehydrated, and sealed with neutral gum. Finally, the pathological changes were observed under the light microscope.

### 2.7 Masson staining

After dewaxing, the sections were stained with hematoxylin for 30 s, followed by distilled water washing and 3‐min treatment with ammonium hydroxide. Afterwards, the sections were immersed in ponceau acid fuchsin stain for 3 min, differentiated with 1% phosphomolybdic acid (2 × 3 min), re‐stained with 1% aniline blue for 5 min, and treated by 1% glacial acetic acid solution for 10 s. Finally, the pathological changes were observed under a light microscope after the sections were sealed.

### 2.8 Immunohistochemistry (IHC)

The sections were dewaxed, washed once with distilled water and thrice with phosphate buffer saline (PBS), and maintained with 3% H_2_O_2_ for 10 min. Subsequently, the sections underwent antigen repair, 20‐min blocking (normal goat serum blocking solution), and incubation with primary antibodies of KLF5 (ab137676, Abcam) and MX1 (#37849, CST) (4°C, overnight). Following thrice PBS washing, the sections were incubated with the secondary antibody for 1 h and then stained with 3,3’‐diaminobenzidine for 1−3 min of color development. At last, the sections were stained with hematoxylin for 3 min, dehydrated, permeabilized, and sealed. Image‐Pro Plus (IPP, Media Cybernetics) software was used to quantify and analyze the positive expression of IHC staining in the sections.

### 2.9 Immunofluorescence (IF)

The paraffin sections were placed at room temperature for 1 h, and washed with PBS (3 × 5 min). Next, the sections were sealed with 10% goat serum for 1 h, and then incubated with the primary antibody against α‐SMA (ab5831, 1:100, Abcam) at 4°C overnight. Afterwards, the primary antibody was recovered and the sections were washed with PBS, followed by the addition of a secondary antibody for 1‐h incubation at 37°C. After PBS washing again, the sections were sealed and observed under a fluorescence microscope, and the images were collected. The relative density of α‐SMA was obtained by IPP6.0 image software.

### 2.10 Cell culture and transfection

Human renal tubular epithelial cell line HK‐2 was purchased from Procell Life Science & Technology Co. Ltd. Cells were cultured in Roswell Park Memorial Institute‐1640 medium added with 10% fetal bovine serum, 100 μ/mL penicillin, and 100 μL/mL streptomycin. Cells were maintained in an incubator containing 5% CO_2_ at 37°C. When the confluence reached 80%, the cells were transfected with si‐KLF5 or cotransfected with si‐KLF5 and oe‐MX1 plasmids using Lipofectamine 3000 kit (Invitrogen). After cell transfection for 24 h, TGF‐β1 (10 ng/mL; PeproTech) was used to induce renal tubule fibrosis in vitro for 24 h.

### 2.11 qRT‐PCR

Peripheral blood mononuclear cells (PBMCs) were isolated from the venous peripheral blood by Ficoll‐Plaque density gradient centrifugation. As per the instructions, total RNA was extracted from PBMCs, renal tissues, or HK‐2 cells with TRIZOL (Invitrogen), and a reverse transcription kit (TaKaRa) was used to conduct reverse transcription. With the help of SYBR Green Master (Roche Diagnostics), qRT‐PCR was carried out on a LightCycler 480 (Roche), with three replicates per reaction. Thermal cycle parameters were as follows: 95°C for 5 min, then 40 cycles of 95°C for 5 s, 60°C for 10 s, and 72°C for 10 s, and extension at 72°C for 5 min. Data analysis was performed using 2^−ΔΔCt^ method (ΔΔCt = experimental group [Ct target gene − Ct internal control] − control group [Ct target gene − internal control]) with glyceraldehyde 3‐phosphate dehydrogenase (GAPDH) serving as the internal control. The primer sequences were synthesized by Sangon Biotechnology Co., Ltd. (see in Table 1).

**Table 1:** Primer sequences used for quantitative reverse transcription‐polymerase chain reaction analysis.

Name of primer	Sequences (5′−3′)
KLF5‐F(Mus)	CACCGGATCTAGACATGCCC
KLF5‐R(Mus)	ACGTCTGTGGAACAGCAGAG
MX1‐F(Mus)	CCCTGAAGGGGATAGGACCA
MX1‐R(Mus)	CCGGCTGTCTCCCTCTGATA
TGF‐β1‐F(Mus)	CCGCAACAACGCCATCTATG
TGF‐β1‐R(Mus)	CTCTGCACGGGACAGCAAT
CTGF‐F(Mus)	AGAACTGTGTACGGAGCGTG
CTGF‐R(Mus)	GTGCACCATCTTTGGCAGTG
KLF5‐F(homo)	ACGCTTGGCCTATAACTTGGT
KLF5‐R(homo)	CTGGTCTACGACTGAGGCAC
MX1‐F(homo)	CTCCGACACGAGTTCCACAA
MX1‐R(homo)	GGCTCTTCCAGTGCCTTGAT
GAPDH‐F(homo)	GAATGGGCAGCCGTTAGGAA
GAPDH‐R(homo)	TCGCCCCACTTGATTTTGGA

*Note*: F, forward; homo, human gene; Mus, mouse gene; R, reverse.

### 2.12 Western blot analysis

Total protein was obtained from renal tissues or cells with Radioimmunoprecipitation Assay lysis buffer, followed by the measurement of concentrations with a protein concentration assay kit (Beyotime). After sodium dodecyl sulfate polyacrylamide gel electrophoresis was performed, the protein was transferred onto a membrane and sealed. Next, the membrane was probed overnight at 4°C with primary antibodies comprising anti‐α‐SMA (19245, CST), anti‐TGF‐β (3711, CST), anti‐CTGF (ab6992, Abcam), anti‐type I collagen (Col I) (91144, CST), anti‐fibronectin (FN) (ab2413, Abcam), and anti‐matrix metalloproteinase 9 (MMP9) (ab283575, Abcam). After Tris‐buffered saline with Tween 20 (TBST) washing (3 × 10 min), the membrane received 2‐h re‐probing with the secondary antibody at room temperature. Another three TBST washes (3 × 10 min), color development, image collection, data analysis were conducted (GAPDH was the internal control).

### 2.13 Dual luciferase reporter gene assay

Based on the previous method,^19^ JASPER was applied to predict the binding site of KLF5 and MX1. Luciferase reporter plasmids of MX1‐WT and MX1‐MUT were constructed and cotransfected into HEK293T cells with oe‐KLF5 or oe‐NC for 48‐h culture (37°C and 5% CO_2_), respectively. Next, the cells were lysed before centrifugation for 3–5 min. The supernatant was collected to determine the luciferase activity using a kit (Dual‐LucifeTRIM24e Reporter Assay System, Promega). The relative luciferase activity was measured with a luminescence detector (Promega Corporation). The parallel experiment was repeated three times.

### 2.14 Statistical analysis

GraphPad Prism 8.0 was utilized for data analysis and the data were expressed as mean ± standard deviation. Data between two groups were compared by the *t*‐test, and the comparison among multiple groups was determined by the one‐way analysis of variance. Post hoc analysis was done using Tukey's multiple comparisons test. A *p* < .05 was defined as statistical significance.

## 3 RESULTS

### 3.1 The high expression of MX1 may be related to renal fibrosis in LN

**Figure 1:**
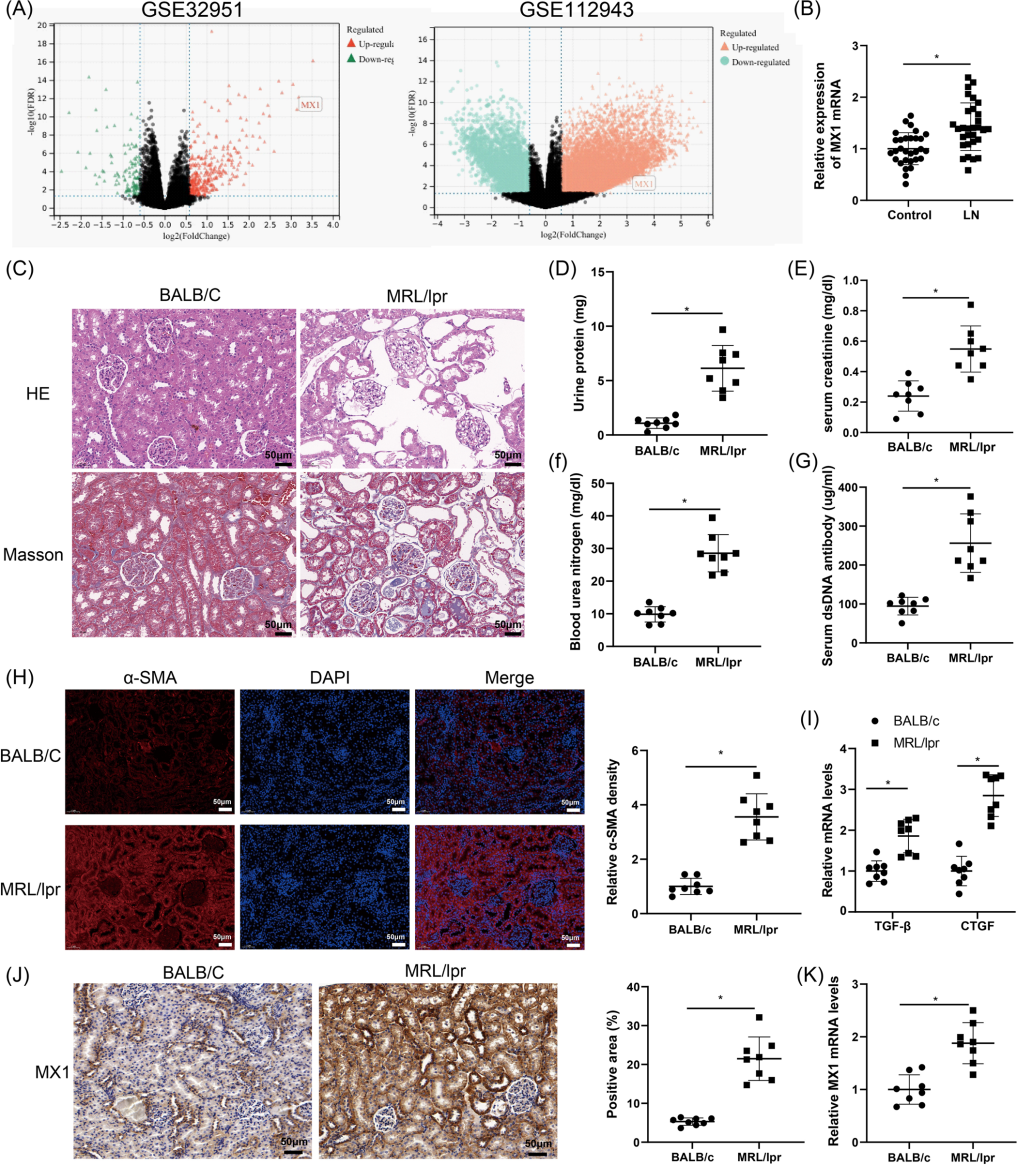
Upregulation of MX1 may be involved in renal fibrosis in LN. *Note*: (A) the volcano plot was used to exhibit the expression of MX1 in LN‐attacked renal tissues. (B) The mRNA level of MX1 in the peripheral blood of LN patients and healthy participants was detected by qRT‐PCR (LN group, *N* = 30, control group, *N* = 30). (C) Pathological changes in renal tissue were observed through H&E and Masson staining (×200). (D–G) The content of urine protein and the levels of serum creatinine, blood urea nitrogen, and serum dsDNA antibody were tested. (H) The expression of α‐SMA in renal tissues of MRL/lpr and BALB/c mice was measured by IF staining (×200). (I) The expression of TGF‐β and CTGF was tested by qRT‐PCR. (J) The expression of MX1 in mouse renal tissues was detected by IHC assay (×200). (K) The mRNA level of MX1 in mouse renal tissues was evaluated by qRT‐PCR. *N* = 8, the *t*‐test was used to assess the comparison between the two groups, *p < .05. dsDNA, double‐stranded DNA; H&E, hematoxylin‐eosin; IF, immunofluorescence; IHC, immunohistochemistry; LN, lupus nephritis; mRNA, messenger RNA; qRT‐PCR, quantitative reverse transcription‐polymerase chain reaction.

First, analysis of GSE32591 and GSE112943 datasets showed that MX1 expression was upregulated in LN tissues versus normal tissues from renal biopsies (Figure 1A). Furthermore, the messenger RNA (mRNA) level of MX1 was higher in the peripheral blood of LN patients than that in the peripheral blood of healthy participants (Figure 1B).

Results from H&E and Masson staining in MRL/lpr mice revealed that the renal structure of mice in the BALB/c group was clear and complete without inflammatory cell infiltration and fibroplasia, while the MRL/lpr group showed obvious proliferation of mesangial cells accompanied by inflammatory cell infiltration and significant collagen deposition in renal tissues (Figure 1C). Compared with the BALB/c group, the content of urine protein and the levels of serum creatinine, blood urea nitrogen, and serum dsDNA were markedly increased in the MRL/lpr group (Figure 1D–G). As displayed by IF staining, the expression of α‐SMA was notably elevated in mouse renal tissues in the MRL/lpr group versus the BALB/c group (Figure 1H). As for the detection of fibrosis marker proteins, results showed that mice in the MRL/lpr group had higher expression of TGF‐β and CTGF than the BALB/c group (Figure 1I). Additionally, results from western blot analysis revealed that, in comparison with the BALB/c group, the expression of α‐SMA, TGF‐β, and CTGF was obviously increased in renal tissues in the MRL/lpr group (Figure S1A), accompanied by the elevated expression of Col I, FN, and MMP9 (Figure S1B). Above data suggested renal injury in LN mice.

To further probe the relationship between MX1 expression and LN, we conducted IHC and qRT‐PCR assays to measure the expression of MX1 in renal tissues. Results manifested that the positive rate and the mRNA level of MX1 were clearly increased in MRL/lpr mice than those in BALB/c mice (Figure 1J,K). Overall, upregulation of MX1 may be involved in renal injury in MRL/lpr mice.

### 3.2 Downregulation of MX1 mitigates renal fibrosis in LN

**Figure 2:**
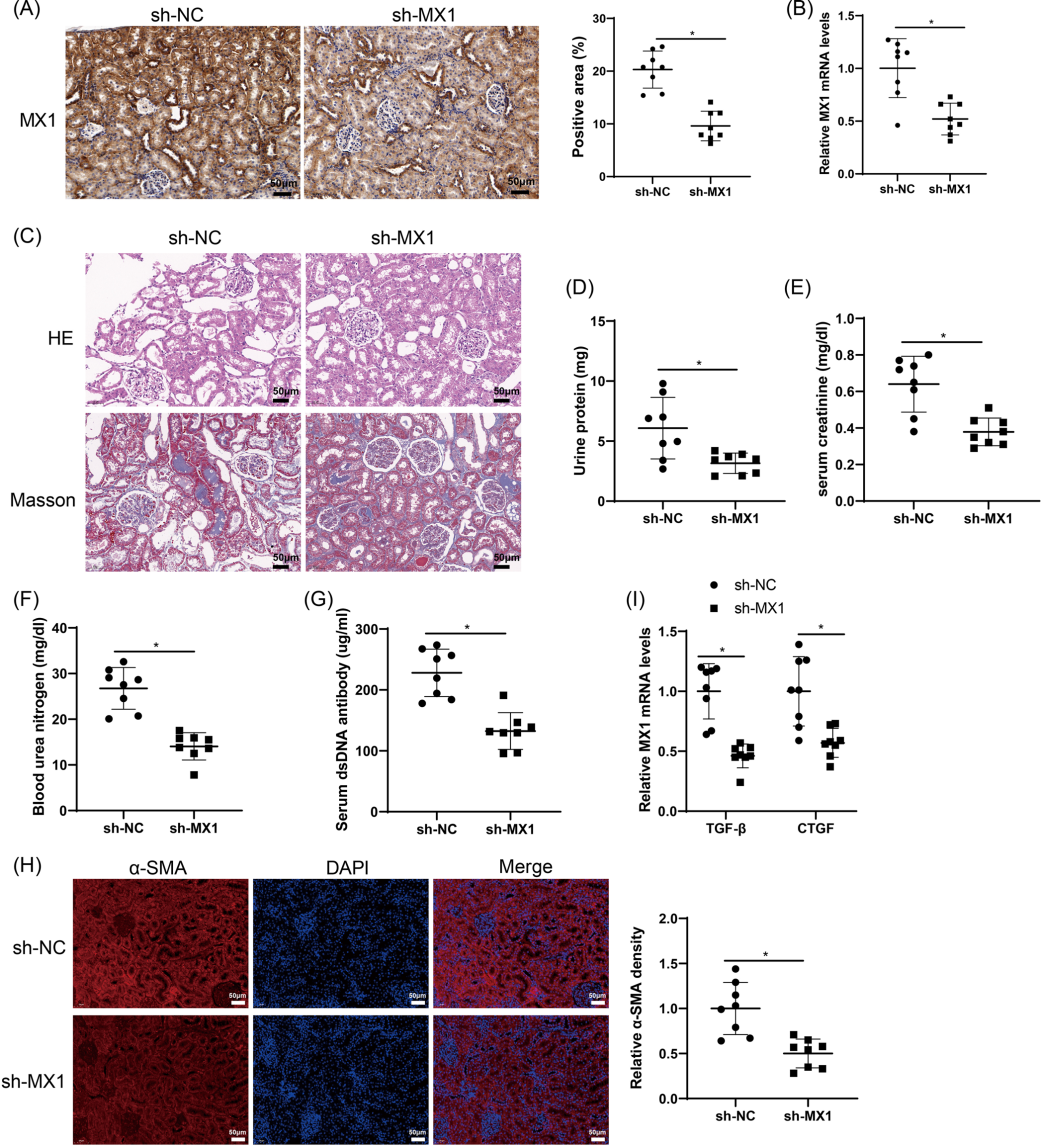
Downregulated MX1 relieves renal fibrosis in MRL/lpr mice. *Note*: After MRL/lpr mice were injected with sh‐MX1. (A) The expression of MX1 in renal tissues was measured by IHC assay (×200). (B) The expression of MX1 in renal tissues was tested by qRT‐PCR. (C) Pathological changes in renal tissue were observed through H&E and Masson staining (×200). (D–G) The levels of urine protein, serum creatinine, blood urea nitrogen, and serum dsDNA antibody were tested. (H) The expression of α‐SMA in renal tissues was measured by IF staining (×200). (G) The expression of TGF‐β and CTGF in renal tissues was tested by qRT‐PCR. *N* = 8, the *t*‐test was used to assess the comparison between the two groups, *p < .05. dsDNA, double‐stranded DNA; H&E, hematoxylin‐eosin; IF, immunofluorescence; IHC, immunohistochemistry; LN, lupus nephritis; qRT‐PCR, quantitative reverse transcription‐polymerase chain reaction.

To deeply explore the effect of MX1 on renal injury, we injected sh‐MX1 into MRL/lpr mice. Results from IHC and qRT‐PCR assays displayed that the sh‐MX1 group had lower positive rate and mRNA level of MX1 compared with the sh‐NC group (Figure 2A,B). In the sh‐MX1 group, mesangial cell proliferation and inflammatory cell infiltration were alleviated and collagen deposition was reduced in MRL/lpr mice (vs. the sh‐NC group) (Figure 2C). In comparison with sh‐NC group, the levels of urine protein, serum creatinine, blood urea nitrogen, and serum dsDNA were dramatically reduced in the sh‐MX1 group (Figure 2D–G). IF staining results showed that α‐SMA expression was evidently decreased in mouse renal tissues of the sh‐MX1 group compared with the sh‐NC group (Figure 2H). Furthermore, the sh‐MX1 group had lower expression of TGF‐β and CTGF versus the sh‐NC group (Figure 2I). Compared with the sh‐NC group, the sh‐MX1 group had reduced expression of α‐SMA, TGF‐β, and CTGF (Figure S1C), AS well as the levels of Col I, FN, and MMP9 (Figure S1D). These results demonstrated that knockdown of MX1 could relieve renal fibrosis and injury in MRL/lpr mice.

### 3.3 KLF5 regulates MX1 transcription

**Figure 3:**
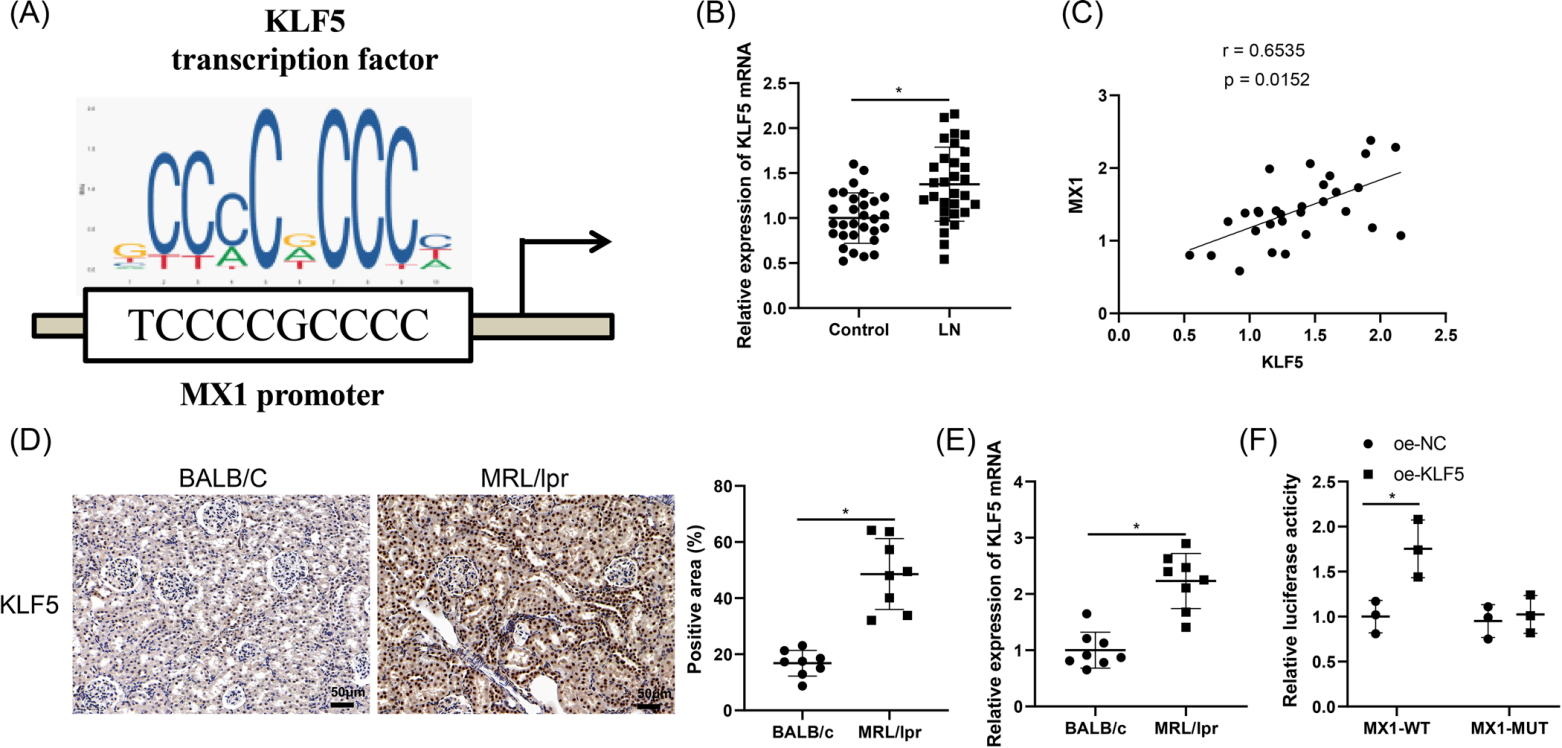
KLF5 modulates MX1 transcription. *Note*: (A) the binding site between KLF5 and MX1 was predicted by JASPER database. (B) The mRNA level of KLF5 in the serum of LN patients and healthy participants was assessed by qRT‐PCR (LN group, *N* = 30, control group, *N* = 30). (C) Pearson coefficient analyzed the correlation between MX1 and KLF5 expression. (D) KLF5 expression was tested by IHC assay (×200). (E) The mRNA expression of KLF5 was examined by qRT‐PCR. (F) The binding relationship between KLF5 and MX1 was detected by double luciferase reporter gene assay. Each cellular experiment was repeated thrice, and animal experiments, *N* = 8. The *t*‐test was applied to measure the comparison between the two groups, *p < .05. IF, immunofluorescence; IHC, immunohistochemistry; mRNA, messenger RNA; qRT‐PCR, quantitative reverse transcription‐polymerase chain reaction.

JASPER database reflected that there was binding sites of KLF5 on MX1 promoter (Figure 3A). Compared with the control group, the mRNA level of KLF5 was evidently increased in the LN group (Figure 3B). Furthermore, Pearson analysis in Figure 3C revealed that MX1 was positively correlated with KLF5 expression. IHC and qRT‐PCR assays demonstrated that the expression of KLF5 in the MRL/lpr group was memorably elevated as compared to the BALB/c group (Figure 3D,E).

Dual luciferase reporter gene assay was further used for verification of the relationship between KLF5 and MX1, which showed that the luciferase activity of the oe‐KLF5 and MX1‐WT cotransfection group was significantly increased than in the oe‐NC and MX1‐WT cotransfection group (Figure 3F). Taken together, the transcription factor KLF5 may modulate renal injury and fibrosis in LN by binding to MX1 promoter.

### 3.4 KLF5 modulates renal fibrosis in LN via MX1

**Figure 4:**
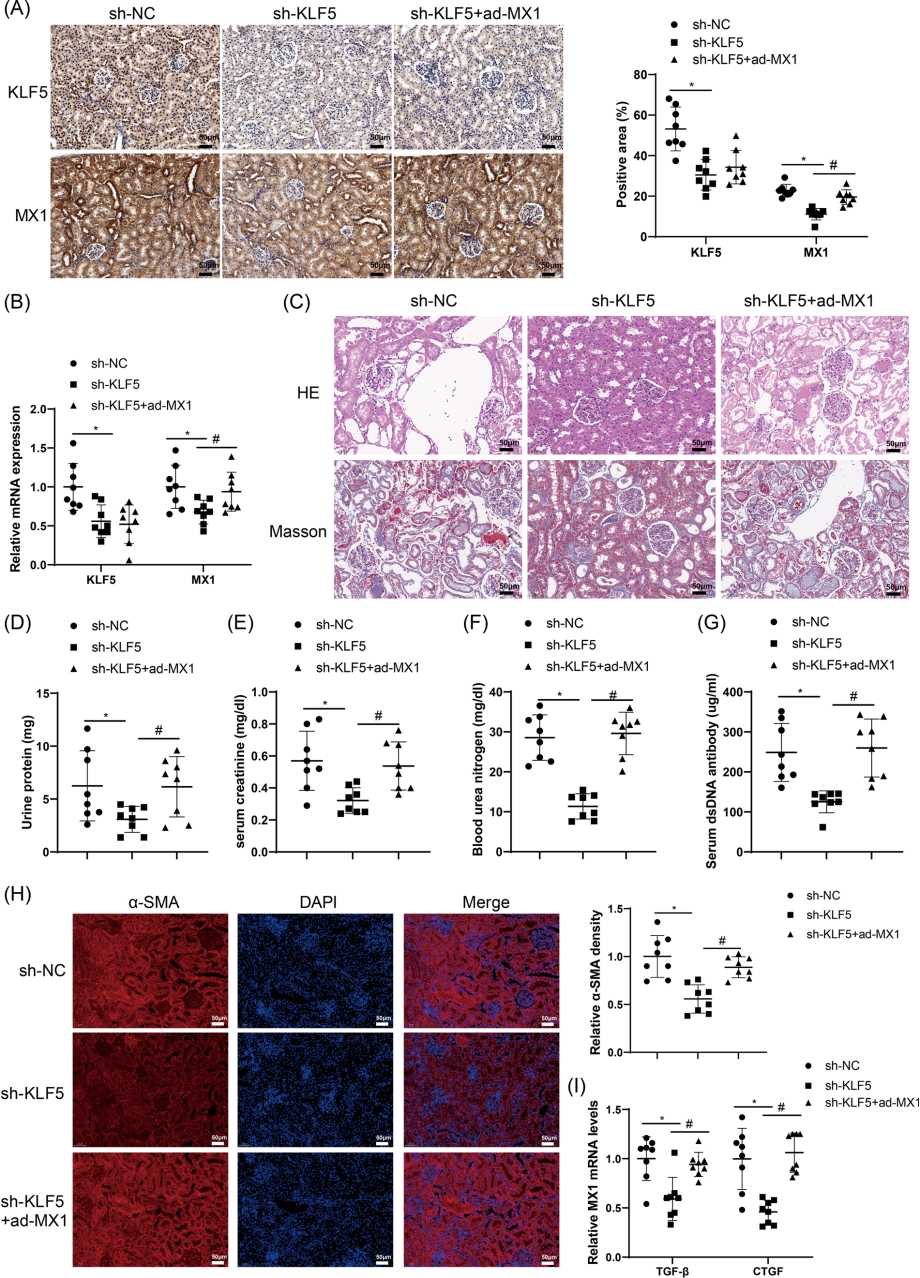
KLF5 regulates renal fibrosis in LN via MX1. *Note*: After MRL/lpr mice were injected with sh‐KLF5 alone or sh‐KLF5 and ad‐MX1. (A) IHC assay was used to detect the expression of KLF5 and MX1 in renal tissues (×200). (B) The mRNA expression of KLF5 and MX1 was measured by qRT‐PCR. (C) Pathological changes in renal tissue were observed through H&E and Masson staining (×200). (D–G) The levels of urine protein, serum creatinine, blood urea nitrogen, and serum dsDNA antibody were examined. (H) the expression of α‐SMA in renal tissues was assessed by IF staining (×200). (I) The expression of TGF‐β and CTGF in renal tissues was tested by qRT‐PCR. *N* = 8, one‐way analysis of variance was employed for comparisons among multiple groups with Tukey's multiple comparisons test used for post hoc analysis, * and ^#^, *p* < .05. dsDNA, double‐stranded DNA; H&E, hematoxylin‐eosin; IF, immunofluorescence; IHC, immunohistochemistry; LN, lupus nephritis; qRT‐PCR, quantitative reverse transcription‐polymerase chain reaction.

sh‐KLF5 alone or sh‐KLF5 and ad‐MX1 was injected into MRL/lpr mice to investigate whether KLF5 could protect against renal tissues through mediating MX1. Results from IHC and qRT‐PCR assays displayed that the sh‐KLF5 group had lower KLF5 and MX1 expression (vs. the sh‐NC group), while in the sh‐KLF5 + ad‐MX1 group, there was no clear difference in KLF5 expression and the expression of MX1 was markedly enhanced (vs. the sh‐KLF5 group) (Figure 4A,B). As depicted in Figure 4C, mesangial cell proliferation and inflammatory cell infiltration were ameliorated and collagen deposition was reduced in MRL/lpr mice of the sh‐KLF5 group compared to the sh‐NC group. In comparison with the sh‐NC group, the levels of urine protein, serum creatinine, blood urea nitrogen, and serum dsDNA were prominently dropped in the sh‐KLF5 group (Figure 4D–G). As expected, the expression of α‐SMA, TGF‐β, and CTGF was visibly decreased in MRL/lpr mouse renal tissues of the sh‐KLF5 group (vs. the sh‐NC group) (Figure 4H,I). However, compared with the sh‐KLF5 group, the renal histopathology and renal dysfunction in the sh‐KLF5 + ad‐MX1 group were aggravated (Figure 4C–I). From the results of western blot analysis, the expression of α‐SMA, TGF‐β, and CTGF was markedly lower than those in the sh‐NC group (Figure S1E), accompanied by markedly downregulated Col I, FN, and MMP9 levels (Figure S1F), whereas simultaneous injection of sh‐KLF5 and ad‐MX1 enhanced the expression of these proteins (vs. the sh‐NC group) (Figure S1E,F). Hence, KLF5 regulated renal fibrosis and injury in LN via MX1.

### 3.5 KLF5 regulates renal fibrosis in TGF‐β1‐induced HK‐2 cells via MX1

**Figure 5:**
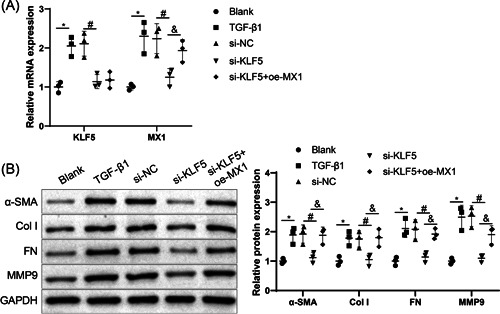
KLF5 modulates renal fibrosis in TGF‐β1‐induced HK‐2 cells via MX1. *Note*: (A) the mRNA expression of KLF5 and MX1 was tested by qRT‐PCR in TGF‐β1‐induced HK‐2 cells. (B) The protein expression of α‐SMA, Col I, FN, and MMP9 was measured by western blot analysis in TGF‐β1‐induced HK‐2 cells. Each cellular experiment was repeated thrice. One‐way analysis of variance was employed for comparisons among multiple groups with Tukey's multiple comparisons test used for post hoc analysis, *, ^#^, and ^&^, *p* < .05. Col I, type I collagen; FN, fibronectin; MMP9, matrix metalloproteinase 9; qRT‐PCR, quantitative reverse transcription‐polymerase chain reaction; TGF, transforming growth factor.

To further explore the biological function of KLF5 and MX1 in the progression of renal fibrosis, we transfected si‐KLF5 or cotransfected si‐KLF5 and oe‐MX1 into HK‐2 cells, followed by 24‐h stimulation with TGF‐β1. Results from qRT‐PCR showed that the mRNA expression levels of KLF5 and MX1 were significantly increased in TGF‐β1 group versus blank group. In comparison with si‐NC group, the mRNA expression of KLF5 and MX1 was dramatically diminished in si‐KLF5 group, while in si‐KLF5 + oe‐MX1 group, increased MX1 mRNA expression was detected compared with the si‐KLF5 group, but KLF5 mRNA expression showed no significant difference between the two groups (Figure 5A). Subsequently, as reflected by western blot analysis, the protein expression of α‐SMA, Col I, FN, and MMP9 was markedly augmented in the TGF‐β1 group (vs. the blank group). Furthermore, transfection of si‐KLF5 obviously inhibited the protein expression of these proteins compared with the si‐NC group, whereas cotransfection of si‐KLF5 and oe‐MX1 reversed that expression pattern as compared to the si‐KLF5 group (Figure 5B). Combining with the above findings, KLF5 could regulate renal fibrosis in TGF‐β1‐induced HK‐2 cells via MX1.

## 4 DISCUSSION

Renal fibrosis is a common pathological feature of end‐stage renal disease and requires renal replacement therapy, which is a huge economic burden worldwide.^20^ To improve the prognosis of LN, it is urgent to identify new therapeutic drugs targeting key mediators of renal fibrosis. In the present study, we investigated the relationship between KLF5 and MX1 in in MRL/lpr mice and TGF‐β1 induced HK‐2 cells. The in vivo and in vitro experiments indicated that the loss of KLF5 relieved renal fibrosis and injury in LN via downregulating the transcription level of MX1.

Currently, routine biomarkers such as urine protein, serum creatinine, serum dsDNA antibody, and other serum complement have been extensively researched in LN.^21^ Increased levels of these markers were detected in the peripheral blood of LN patients and in the renal tissues of MRL/lpr mice. In addition, we also detected increased expression of renal fibrosis related proteins (α‐SMA, TGF‐β, CTGF, Col I, FN, and MMP9). Coincidentally, Wei et al.^22^ demonstrated that the levels of these fibrosis‐related proteins were obviously increased in cisplatin‐injured HK‐2 cells and renal tissues from rats. Combined with pathological changes in renal tissues, we concluded that renal tissues were injured in LN mice. In the progression of LN, interferon response was irritated in most kidney cells.^23^ Interestingly, cumulative evidence suggested the correlation between interferon‐related genes and LN‐induced injury in kidney cells. miR‐130b overexpression was reported to suppress type Ⅰ interferon pathway in primary renal mesangial cells by downregulating interferon regulatory factor 1 and reduce urine protein, complex deposition, and glomeruli lesion in LN.^24^ The 35‐kDa interferon‐induced protein (IFP35) was recently demonstrated to be upregulated in LN ^25^ and acted as a promoting role in inflammatory response and apoptosis of glomerular cells.^26^ Interferon‐inducible MX1 plays an important role in some autoimmune diseases.^27^ MX1 expression was measured to be upregulated in SLE patients.^28^ Analysis of datasets in GEO database showed that MX1 was upregulated in LN tissues compared with normal tissues from renal biopsies. Subsequent functional experiments revealed that the mRNA level of MX1 was increasingly expressed in renal tissues and downregulation of MX1 relieved renal fibrosis and injury in MRL/lpr mice. Similarly, the expression of MX1 was elevated in the kidney of LN patients before treatment, and decreased after immunosuppressive treatment.^29^ A previous work showed that circular RNA 0007059 could restore the viability and repress inflammation in renal mesangial cells and HEK293 cells and concomitantly downregulate the expression of interferon‐inducible genes (CXCL10, IFIT1, ISG15, and MX1).^30^ Remarkably, type Ⅰ interferon pathway was previously reported to contribute to tissue fibrosis in systemic sclerosis.^31^ These data suggested that MX1 was closely associated with the occurrence of renal injury and fibrosis in LN. However, the specific regulatory mechanism of MX1 in renal injury in LN has rarely been reported.

Based on JASPER database, we found that there were binding sites between KLF5 and MX1 promoter region, and luciferase reporter assay and coefficient analysis further identified their binding relationship. Moreover, KLF5 was assessed to be increased in renal tissues of MRL/lpr mice and the peripheral blood of LN patients. Reportedly, KLF5 could regulate fibrosis in a variety of organs, including kidney,^14^ heart,^32^ liver,^33^ and lung.^34^ Chen et al.^35^ demonstrated that KLF5 was expressed in tubular cells and associated with the pathogenesis of renal fibrosis in fibrotic kidneys. Knockdown of KLF5 markedly diminished the expression of TGF‐β1, α‐SMA, and collagen‐1, ultimately attenuating fibrotic lesions.^36^ In addition, KLF5 has been identified as a transcription factor of interferon‐induced transmembrane proteins in human alveolar basal epithelial cells to protect against H5N1 virus infection.^37^ Our data verified that downregulation of KLF5 alone improved renal injury and fibrosis in MRL/lpr mice, accompanied by declined levels of urine protein, serum creatinine, blood urea nitrogen, serum dsDNA, and fibrosis‐related proteins. However, overexpression of interferon‐inducible MX1 boosted renal histopathology and renal dysfunction in the presence of KLF5 suppression. Subsequently, we conducted in vitro experiments to further confirm the results of the in vivo experiment by inducing HK‐2 cell fibrosis with TGF‐β1.

## 5 CONCLUSION

In conclusion, our study elucidated the protective effect of the loss of KLF5 by inhibiting the transcription level of MX1 in LN‐induced renal fibrosis and injury. To our knowledge, this is the first evidence for the regulatory mechanism of KLF5 and MX1 in LN‐triggered renal injury. The results of our experiments may provide a novel avenue in the field of prevention and management of renal injury in LN. Additionally, we only used female mice in this study, so the obtained results were not fully representative of all mice. Therefore, further research with both female and male mice is warranted in the future.

## AUTHOR CONTRIBUTIONS

**Shanshan Tao**: conceived the ideas (lead); designed the experiments (lead); performed the experiments (lead); analyzed the data (lead); provided critical materials (lead); wrote the manuscript (lead). **Xiao Tan**: performed the experimentsm (equal); analyzed the data (equal); wrote the manuscript (equal). **Wen Chai**: performed the experiments (equal); analyzed the data (equal). **Xiaojie Peng**: performed the experiments (equal). **Weimin Zheng**: analyzed the data (equal); wrote the manuscript (equal). **Rui Fu**: wrote the manuscript (equal). **Meihui Deng**: conceived the ideas (equal); designed the experiments (equal); provided critical materials(equal); supervised the study (lead).

## CONFLICT OF INTEREST STATEMENT

The authors declare no conflicts of interest.

## Supporting information


**Supplementary figure 1** Detection of the expression of fibrosis‐related proteins with western blot analysis. **Notes**: A‐F, the protein expression of α‐SMA, TGF‐β, CTGF, Col I, FN, and MMP9 in mouse renal tissues was assessed by western blot analysis, N = 8. The t‐test was applied to measure the comparison between the two groups, and one‐way analysis of variance was employed for comparisons among multiple groups with Tukey's multiple comparisons test used for post hoc analysis, * and ^#^, *P* < 0.05. Col I, type I collagen; FN, fibronectin; MMP9, matrix metalloproteinase 9.

## Data Availability

The datasets used or analyzed during the current study are available from the corresponding author on reasonable request.
