# Supplementary figures and images for: Knockdown of KLF5 ameliorates renal fibrosis in MRL/lpr mice via inhibition of MX1 transcription
Source: Immun Inflamm Dis. 2023 Jul 27;11(7):e937. doi: 10.1002/iid3.937 (PMC10373570; doi:10.1002/iid3.937)

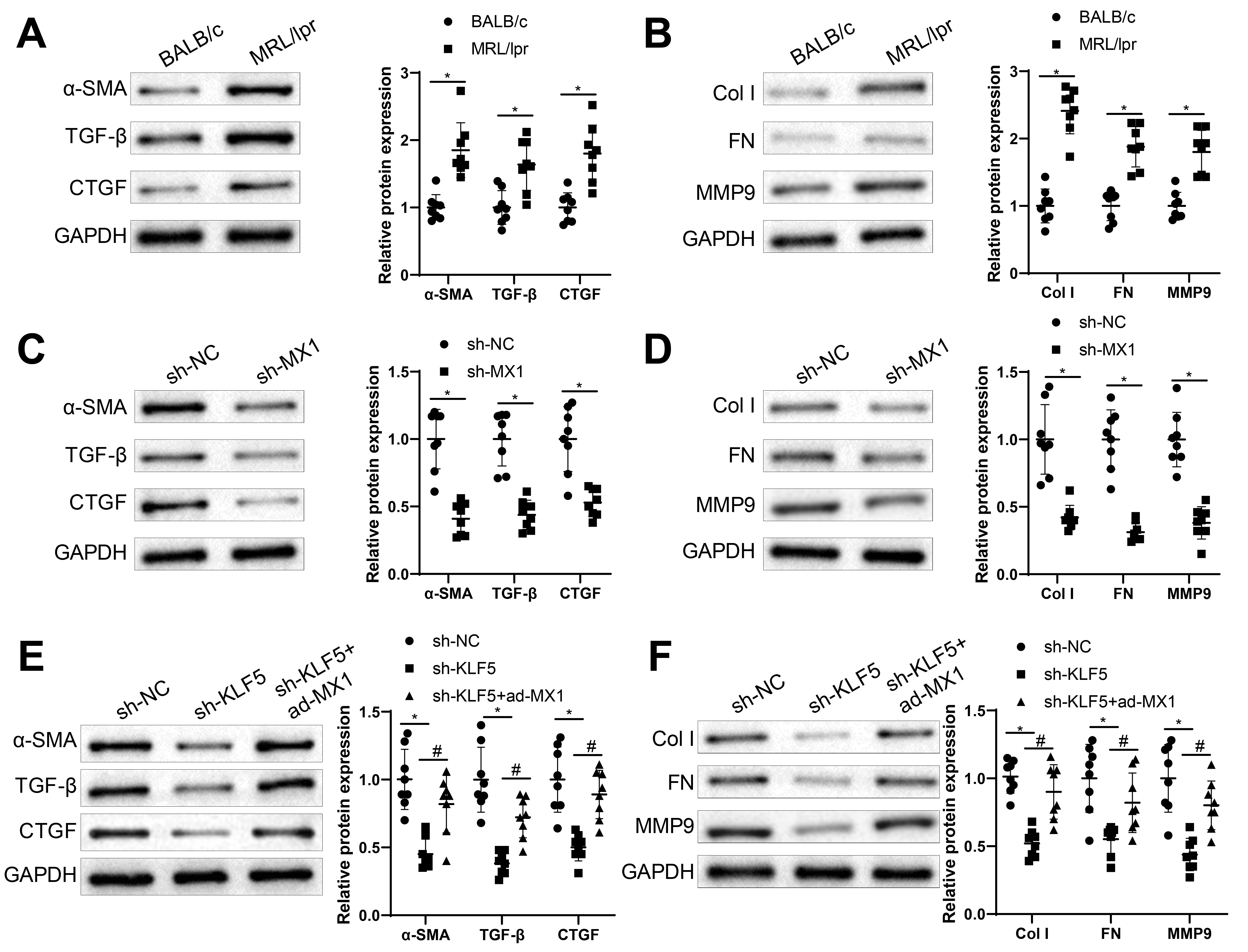

Supplement: Supplementary file 1 — Supplementary figure 1 Detection of the expression of fibrosis‐related proteins with western blot analysis. Notes: A‐F, the protein expression of α‐SMA, TGF‐β, CTGF, Col I, FN, and MMP9 in mouse renal tissues was assessed by western blot analysis, N = 8. The t‐test was applied to measure the comparison between the two groups, and one‐way analysis of variance was employed for comparisons among multiple groups with Tukey's multiple comparisons test used for post hoc analysis, * and #, P < 0.05. Col I, type I collagen; FN, fibronectin; MMP9, matrix metalloproteinase 9. [file IID3-11-e937-s001.jpg]
